# Supplementary material for: Cationic Magnetite Nanoparticles for Increasing siRNA Hybridization Rates
Source: Nanomaterials (Basel). 2020 May 27;10(6):1018. doi: 10.3390/nano10061018 (PMC7352639; doi:10.3390/nano10061018)
Supplement: Supplementary file 1 [file nanomaterials-10-01018-s001.pdf]

nanomaterials

Supplementary Materials

Cationic Magnetite Nanoparticles for Increasing siRNA Hybridization Rates

Artur Y. Prilepskii, Arseniy Y. Kalnin, Anna F. Fakhardo, Elizaveta I. Anastasova, Daria D. Nedorezova, Grigorii A. Antonov and Vladimir V. Vinogradov *

International Institute "Solution Chemistry of Advanced Materials and Technology", ITMO University, 197101 St. Petersburg, Russia; prilepskii@scamt-itmo.ru (A.Y.P.); kalnin@scamt-itmo.ru (A.Y.K.); fakhardo@scamt-itmo.ru (A.F.F.); anastasova@scamt-itmo.ru (E.I.A.); nedorezova@scamt-itmo.ru (D.D.N.); antonov@scamt-itmo.ru (G.A.A.)

* Correspondence: vinogradov@scamt-itmo.ru

1. Materials and Methods

1.1. Chemicals

Iron (II) chloride tetrahydrate (≥98.5%), iron (III) chloride hexahydrate (≥99%), sodium citrate, PEG 8k, gold (III) chloride solution (99.99%, 30 wt.% in dilute HCl), sodium borohydride (98%, powder), cetyltrimethylammonium bromide (99.0%, BioUltra, for molecular biology), SYBR Gold, acrylamide, bisacrylamide, urea, tris base, boric acid, ethylenediaminetetraacetic (disodium salt), tetramethylethylenediamine (TEMED) ammonium persulfate and PBS were purchased from Sigma-Aldrich (St. Louis, MO, USA). The deionized water was from Elix Essential 3UV, Millipore (Burlington, MA, USA). Sodium silicate, acetic acid and acridine orange were obtained from Chimmed (Moscow, Russia). The oligonucleotides used in the study were ordered in the IDT (Coralville, IA, USA), dissolved in RNAse/DNAse free water to a final concentration of 100 μM and were stored frozen. Before the experiments, stocks were dissolved to 10 μM in RNAse/DNAse free water.

The oligonucleotides sequences used in the study were:

DAD1_sense CCACACCGCAGCGUCUGAAUU and

DAD1_antisense UUCAGACGCUGCGGUGUGGGA.

1.2. Characterization Techniques

The crystal phase and crystallinity of the samples were studied by X-ray diffraction method (Rigaku SmartLab 3 diffractometer of the Engineering center of the Saint-Petersburg State Technological Institute (Technical University)) using Cu-Kα irradiation (λ = 1.54 Å). The samples were scanned along 2θ in the range of 5–80° at a speed of 0.5°/min. For XRD analysis, the samples were dried at 120 °C for 4 h. The particle size and zeta potential of the NPs were measured using a Photocor Compact Z (Photocor, Moscow, Russia). For SEM analysis, the samples were dried in vacuo for 1 h and examined using a Tescan VEGA 3 electron microscope (Tescan, Brno, Czech Republic).

1.3. Fluorescent Measurements

Fluorescence measurements with acridine orange were performed by the following method. Three different concentrations of GNPs-15 were prepared: the stock solution, the stock solution diluted 10 times, and the stock solution diluted 100 times. 1 μL of acridine orange (0.5 μg/mL) was mixed with different volumes of these three GNPs solutions, namely, 1, 2, and 5 μL (an additional sample with 10 μL was made for the stock solution). The whole volume of the probes was brought to 100 μL with water. These probes were used as reference values. For probes with siRNA, 1 μL of S and As siRNA solutions (1 μM) were added. All probes were left at room temperature for 30 minutes,

and fluorescence was measured on a CaryEclipse spectrofluorimeter (Santa Clara, CA, USA) at the excitation wavelength 490 nm and the emission wavelength 525 nm.

1.4. Analysis of Gel Images

Gel pictures were analyzed as follows: Each band was saved as a separate image of the same size in pixels, and the total intensity of pixels was calculated using Wolfram Mathematica software with in-build function ImageData. The hybridization rate (HR_t) of siRNA at the specified time t was calculated as a ratio of the value of mean upper band intensity (hybridized siRNA) to mean total intensity (hybridized and non-hybridized siRNA) and expressed in percentage:

$$\mathrm{HR}_t = 100 \times \frac{\frac{1}{\mathrm{n}} \times \sum_1^{\mathrm{n}} A_n^t}{\frac{1}{\mathrm{n}} \times \sum_1^{\mathrm{n}} (A_n^t + B_n^t)} \quad (1)$$

where A_n^t is the brightness of the *n*th upper band (hybridized siRNA) at time *t*, and B_n^t is the brightness of the nth lower band (non-hybridized siRNA) at time *t*. $\frac{1}{\mathrm{n}} \times \sum_1^{\mathrm{n}} A_n^t$ is the mean intensity of *n* upper bands (for example, in Figure 4B, the mean intensity of upper bands of lanes 1–5 for experimental samples), and $\frac{1}{\mathrm{n}} \times \sum_1^{\mathrm{n}} (A_n^t + B_n^t)$ is the mean value of a sum of upper and lower band intensities. For all experiments $n = 5$. An example of the calculation along with raw data is presented in this document.

1.5. Statistical Analysis

Data were processed using conventional methods of variation statistics. Differences between groups were considered significant based on the Student's *t*-test.

2. Results

2.1. Scanning Electron Microscopy Investigation

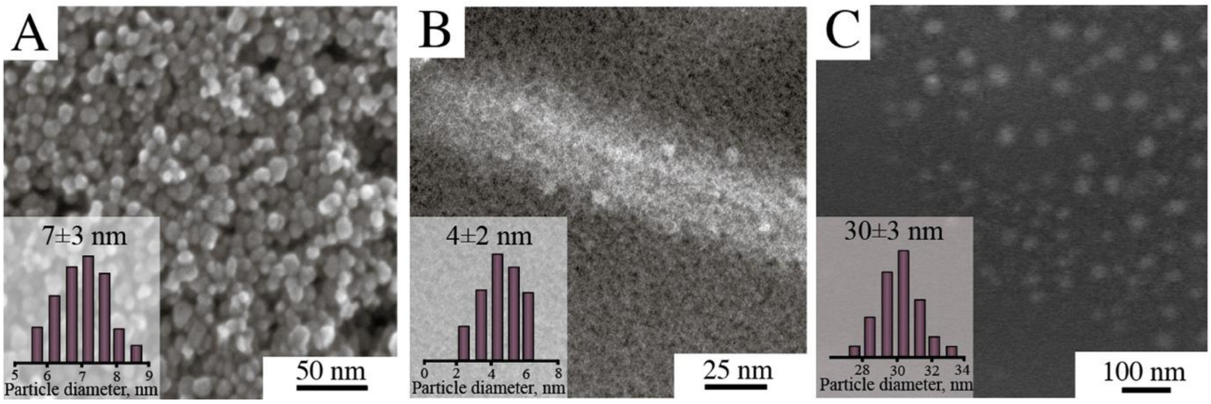

Figure S1. SEM images and particle size distribution of NPs: (**A**) magnetite nanoparticles (MNPs); (**B**) silica nanoparticles (SNPs); and (**C**) gold nanoparticles with average diameter 30 nm (GNP-30).

2.2. Surface Investigation of MNPs

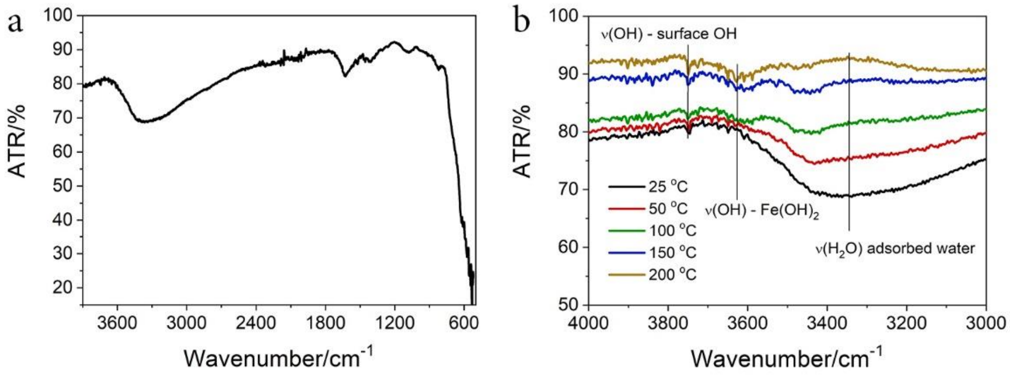

Figure S2. Attenuated total reflection infrared spectra (IR-ATR) of MNPs: (**a**) FTIR spectra of MNPs; and (**b**), water removal at elevated temperatures, reveals the presence of OH-groups on the surface of MNPs.

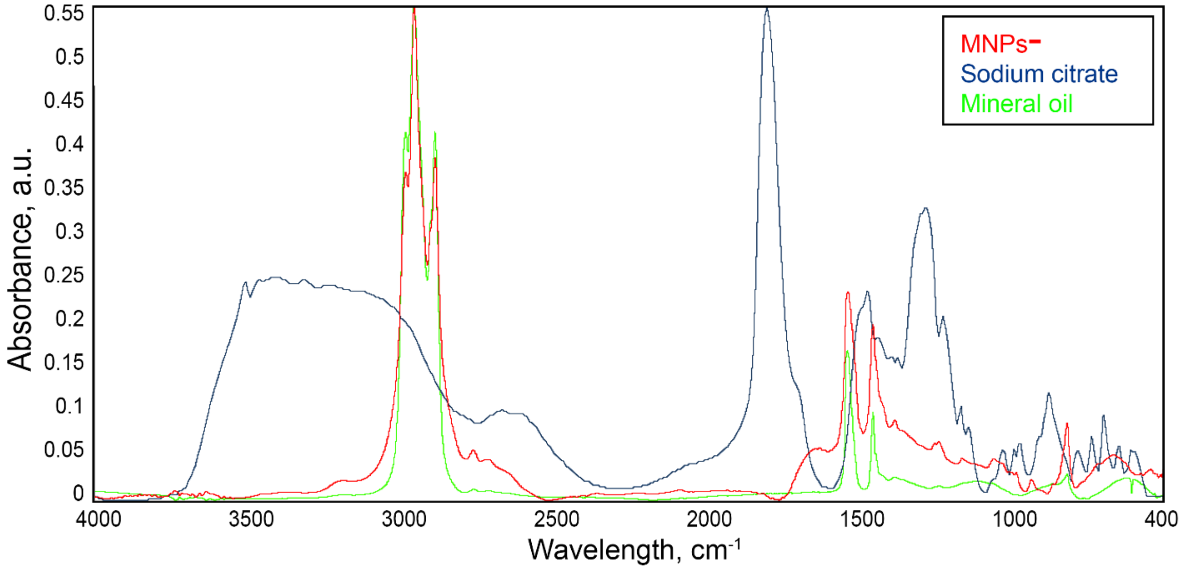

Figure S3. Fourier-transform infrared spectroscopy (FTIR) spectra of MNPs$^-$, sodium citrate, and the embedding medium (mineral oil).

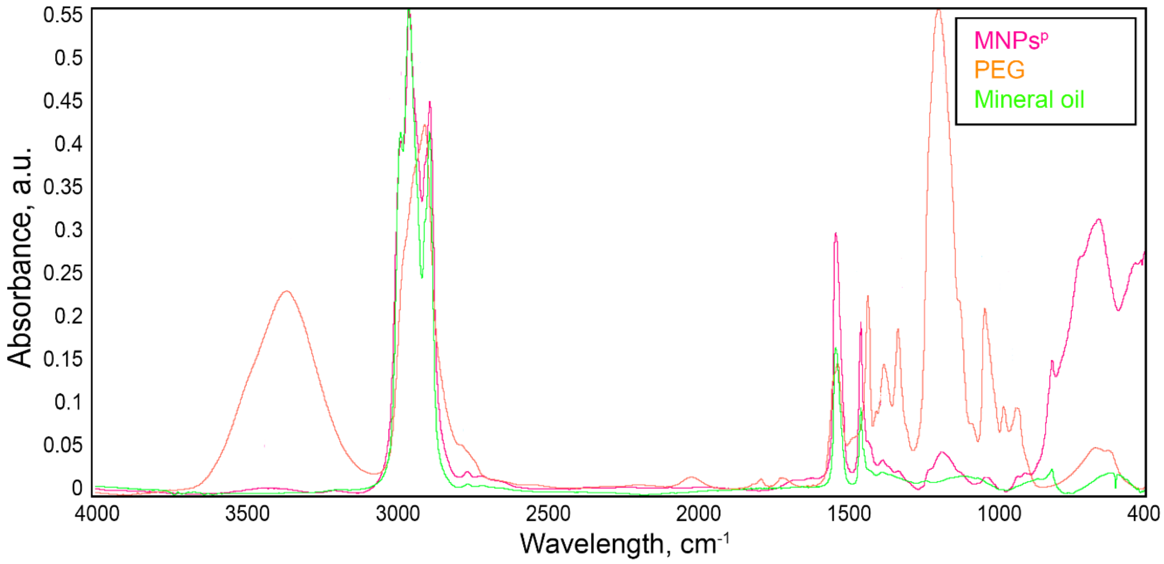

Figure S4. FTIR spectra of $MNPs^P$, polyethylene glycol (PEG), and the embedding medium (mineral oil).

2.3. *Evaluation of RNA Hybridization Level Using Polyacrylamide Gel Electrophoresis (PAGE)*

Tables S1–S6 present intensity values for greyscale images of PAGE bands for all three types of NPs. Each pixel in the image has a maximum intensity value of 255 (entirely white) and a minimum value of 0 (entirely black). The presented numbers are the total intensity for each band image.

Table S1. The intensity of upper bands of PAGE with MNPs.

		**0 hours**	**0.5 hours**	**1 hour**	**2 hours**	**3 hours**
Sample	1st repeat	523,775	374,411	459,844	508,515	507,921
	2nd repeat	583,958	544,730	537,486	497,084	444,736
	3rd repeat	475,494	554,196	526,041	483,147	466,080
	4th repeat	337,224	224,971	381,961	484,059	442,288
	5th repeat	335,349	244,931	328,545	496,135	432,326
Control	1st repeat	137,200	216,452	216,731	332,501	248,591
	2nd repeat	178,349	226,702	259,824	295,946	265,834
	3rd repeat	188,402	168,019	310,895	209,266	253,189
	4th repeat	160,027	371,901	385,230	198,782	250,918
	5th repeat	200,109	254,324	354,186	172,435	300,775

Table S2. The intensity of lower bands of PAGE with MNPs.

		**0 hours**	**0.5 hours**	**1 hour**	**2 hours**	**3 hours**
Sample	1st repeat	282,956	188,517	206,509	215,791	213,525
	2nd repeat	245,673	210,075	240,947	218,636	186,241
	3rd repeat	247,727	231,569	241,352	22,034	195,533
	4th repeat	149,618	139,973	156,357	199,658	176,139
	5th repeat	151,462	125,028	128,544	197,878	16,779
Control	1st repeat	133,568	974,808	683,894	109,237	753,894
	2nd repeat	125,713	107,833	817,506	883,631	778,227
	3rd repeat	117,052	100,681	118,392	452,682	869,075
	4th repeat	126,156	139,516	143,855	507,992	907,851
	5th repeat	147,081	13,808	126,496	578,839	102, 776

Table S3. The intensity of upper bands of PAGE with GNPs.

		0 hours	0.5 hours	1 hour	2 hours	3 hours
Sample	1st repeat	509,775	237,431	334,499	282,619	-*
	2nd repeat	450,685	232,524	158,976	276,353	385,944
	3rd repeat	354,136	231,304	268,708	294,620	146,882
	4th repeat	316,964	161,380	239,052	188,495	243,178
	5th repeat	284,848	216,760	295,008	186,802	261,078
Control	1st repeat	381,938	202,087	179,695	-*	362,799
	2nd repeat	288,132	163,805	266,852	181,238	205,475
	3rd repeat	255,712	197,399	142,436	244,729	231,473
	4th repeat	431,977	189,007	216,356	271,378	370,446
	5th repeat	433,606	223,594	193,334	-*	398,841

*Note: samples for these bands were lost during preparation.

Table S4. The intensity of lower bands of PAGE with GNPs.

		0 hours	0.5 hours	1 hour	2 hours	3 hours
Sample	1st repeat	257,958	128,913	176,819	142,038	-*
	2nd repeat	214,475	123,551	963,871	159,678	197,465
	3rd repeat	183,594	108,355	118,692	105,964	813,278
	4th repeat	126,866	525,004	623,259	679,561	703,769
	5th repeat	138,736	791,004	103,761	651	835,502
Control	1st repeat	154,866	949,325	734,569	-*	103,084
	2nd repeat	179,256	669,271	951,757	885,678	797,659
	3rd repeat	135,361	87,289	12,819	107,764	882,765
	4th repeat	209,898	107,369	845,863	926,776	172,212
	5th repeat	281,035	113,729	835,467	-*	201,396

*Note: samples for these bands were lost during preparation.

Table S5. The intensity of upper bands of PAGE with SNPs.

		0 hours	0.5 hours	1 hour	2 hours	3 hours
Sample	1st repeat	295,494	331,319	230,222	239,579	171,976
	2nd repeat	244,876	335,565	232,102	241,896	178,953
	3rd repeat	288,444	275,894	196,235	228,515	176,680
	4th repeat	260,855	291,089	163,298	206,760	178,547
	5th repeat	246,250	285,301	168,098	190,354	147,750
Control	1st repeat	246,734	210,603	197,187	212,898	158,964
	2nd repeat	274,491	219,515	179,632	210,999	168,215
	3rd repeat	227,573	301,675	192,034	236,974	140,436
	4th repeat	285,854	263,208	186,476	254,187	154,427
	5th repeat	295,250	221,912	207,633	282,340	-*

*Note: samples for these bands were lost during preparation.

Table S6. The intensity of lower bands of PAGE with SNPs.

		0 hours	0.5 hours	1 hour	2 hours	3 hours
Sample	1st repeat	145,314	187,057	150,034	140,371	14,407
	2nd repeat	144,564	177,381	133,938	172,008	169,951
	3rd repeat	137,139	135,998	128,687	116,596	152,905
	4th repeat	12,724	110,835	105,899	118,478	13,756
	5th repeat	107,163	134,465	112,708	104,366	122,197
Control	1st repeat	107,334	110,678	113,188	102,405	126,898
	2nd repeat	132,227	140,177	112,562	114,873	12,534
	3rd repeat	139,218	15,004	904,812	135,424	147,015
	4th repeat	159,239	136,976	751,996	146,499	172,095
	5th repeat	149,619	971,043	117,879	131,167	-*

*Note: samples for these bands were lost during preparation.

Below are examples of the calculation of hybridization rates (HR) for MNPs after 0 hours of incubation.

The HR for samples with MNPs (lanes 1–5 as in Figure 4) was 0.6767:

$$HRs = \frac{\frac{1}{5}(5237.75 + 5839.58 + 4754.94 + 3372.24 + 3353.49)}{\frac{1}{5}\big((5237.75 + 2829.56) + (5839.58 + 2456.73) + (4754.94 + 2477.27) + (3372.24 + 1496.18) + (3353.49 + 1514.62)\big)}$$

The HR, for control (HRc) (lanes 6–10 as in Figure 4) gave the value 0.5708

$$HRc = \frac{\frac{1}{5}(1372 + 1783.49 + 1884.02 + 1600.27 + 2001.09)}{\frac{1}{5}((1372 + 1335.68) + (1783.49 + 1257.13) + (1884.02 + 1170.52) + (1600.27 + 1261.56) + (2001.09 + 1470.81))}$$

The total increase in HR was calculated as follows:

$$\Delta HR = \frac{HRs}{HRc} \times 100 \tag{2}$$

This gave $\Delta HR = 118.5\%$, i.e., the sample with MNPs had an 18.5% better hybridization rate in comparison with the control.

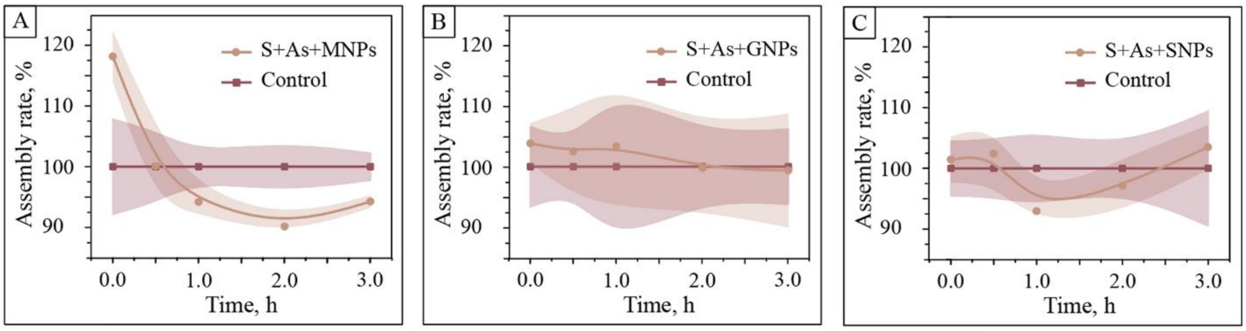

Figure S5. Hybridization rates in systems with (**a**) MNPs, (**b**) GNPs, and (**c**) SNPs over time.

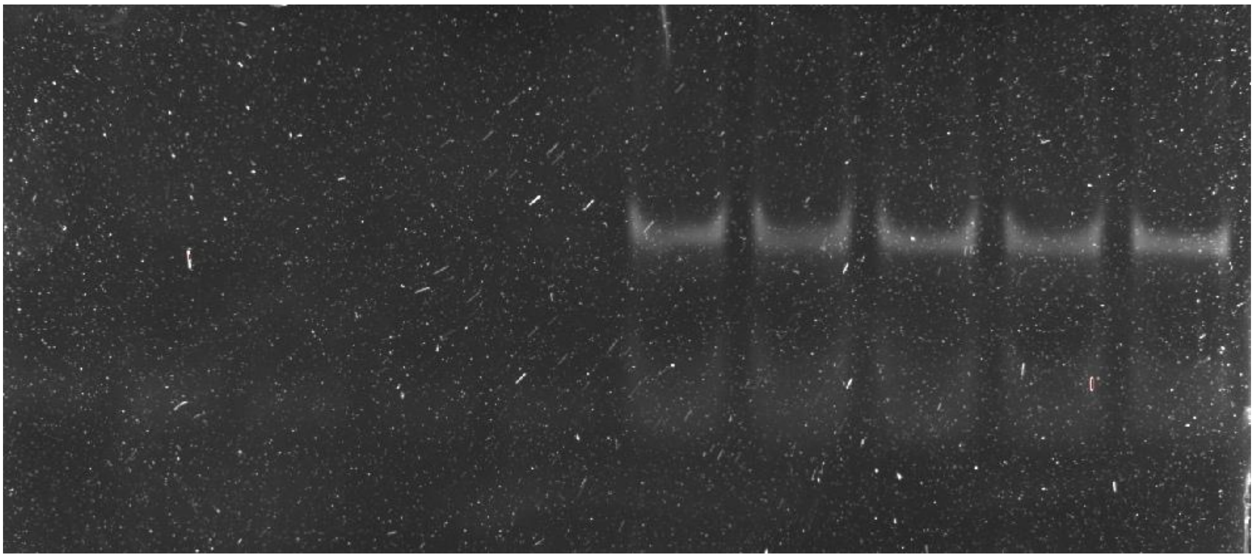

Figure S6. PAGE with the sample incubated with GNPs-15 (lanes 1–5) and the control without NPs (lanes 6–10). No siRNA was detected in the first five lanes.

2.4. Synthesis of Nanoparticles

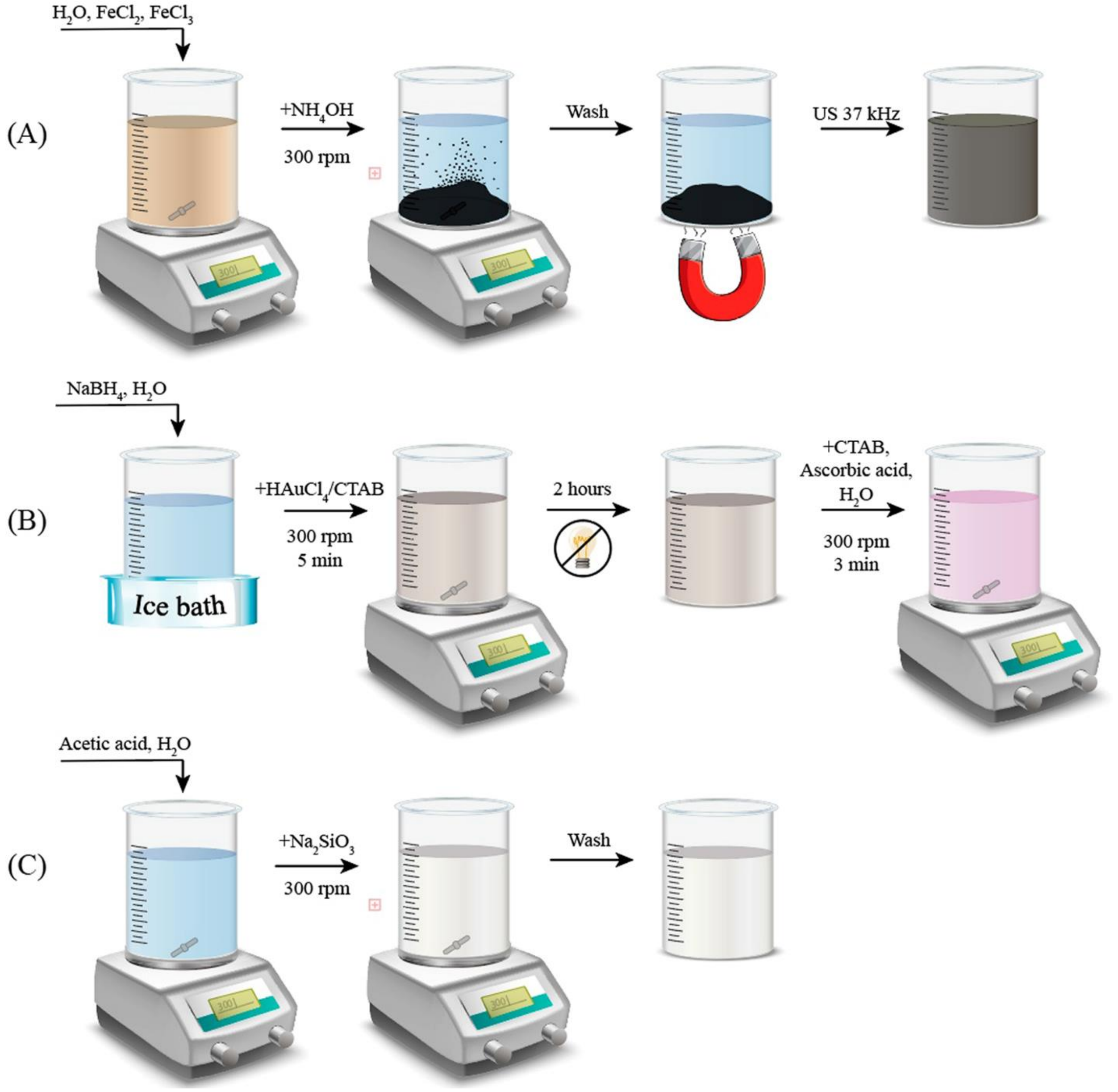

Figure S7. The synthesis scheme of nanoparticles. (**a**) MNPs: Iron salts were dispersed in water followed by the addition of ammonium hydroxide. Black precipitate was then collected by a magnet, washed until neutral pH with water and subjected to ultrasound for 4 hours. (**b**) GNPs: Ice-cold sodium borohydride was mixed with tetrachloroauric acid and CTAB, and left in the dark for seed growth. Next, CTAB and ascorbic acid were added to form GNPs. (**c**) SNPs: A water solution of acetic acid was mixed with sodium metasilicate following the formation of SNPs.

2.5. Hybridization Rates for the Second Scheme with MNPs

Sense and antisense sequences were added to the NPs solution in two different ways. First scheme: NPs were added to premixed S and As sequences ((S + As) + NPs). Second scheme: Sense and antisense strands were separately mixed with NPs followed by mixing of (S + NPs) and (As + NPs) complexes together. Table 7S shows the presented data for hybridization rates with MNPs after 0 hours of incubation. Contrary to the 18% increase of HR in the first scheme, here we observed reduction of HR by 4%. We speculated that this occurs due to loss of spatial mobility of siRNA when they first bind to the surface on MNPs.

Table S7. The intensity of PAGE bands with MNPs according to the second scheme of mixing.

		Upper band	Lower band
Sample	1st repeat	963,333	807,725
	2nd repeat	100,824	847,031
	3rd repeat	951,882	817,043
	4th repeat	886,725	763,588
	5th repeat	908,353	763,627
Control	1st repeat	101,173	785,235
	2nd repeat	961,247	745,902
	3rd repeat	986,918	794,608
	4th repeat	106,544	859,996
	5th repeat	-*	-*

*Note: samples for these bands were lost during preparation.

2.6. Correlation Between Greyscale Image Intensity and siRNA Concentration

To prove, that PAGE can be used as a technique for siRNA concentration analysis, we performed a series of dilutions of hybridized siRNA in the range from 4 µM to 0.008 µM and stained it with SYBR Gold, according to protocol. As a result, we found that the correlation between siRNA concentration and fluorescence intensity is linear. At the lowest concentrations dependency becomes not linear, but this is due to the detection limit of this technique.

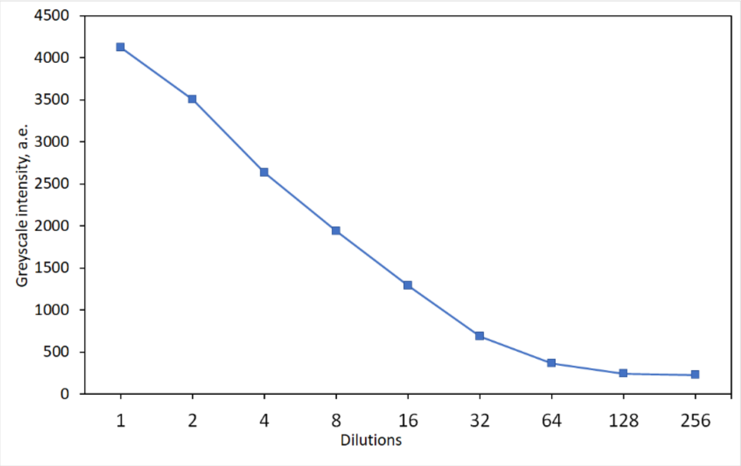

Figure S8. Correlation between fluorescent intensity (in terms of grayscale intensity) and concentration of siRNA.
